# Dental caries is negatively correlated with body mass index among 7-9 years old children in Guangzhou, China

**DOI:** 10.1186/s12889-016-3295-3

**Published:** 2016-07-26

**Authors:** Jing-jing Liang, Zhe-qing Zhang, Ya-jun Chen, Jin-cheng Mai, Jun Ma, Wen-han Yang, Jin Jing

**Affiliations:** 1 Department of Maternal and Child Health, School of Public Health, Sun Yat-Sen University, Guangzhou, 510080 People’s Republic of China; 2 Department of Nutrition and Food Hygiene, Guangdong Provincial Key Laboratory of Tropical Disease Research, School of Public Health, Southern Medical University, Guangzhou, 510515 China; 3 Guangzhou Health Care Clinics of Middle and Primary Schools, Guangzhou, 510080 China; 4 Institute of Child and Adolescent Health, Public Health of Peking University, Beijing, 100191 China

**Keywords:** Body Mass Index, dmft, Dental caries, School children

## Abstract

**Background:**

Evidence linking caries in primary dentition and children’s anthropometric measures is contradictory. We aimed to evaluate the prevalence of primary dental caries and its relationship with body mass index (BMI) among 7-9 years old school children in urban Guangzhou, China.

**Methods:**

This cross-sectional study enrolled 32,461 pupils (14,778 girls and 17,683 boys) aged 7-9 years from 65 elementary schools in Guangzhou. Dental caries was detected according to criteria recommended by the World Health Organization (WHO). The total mean decayed, missing or filled teeth (dmft) of primary dentition were assessed. Weight and height were measured and BMI was calculated. Children were classified into underweight, normal weight, overweight and obesity groups by BMI based on Chinese criteria. Z-score of BMI-for-age (BAZ) was calculated by WHO standardized procedure. Multivariable odds ratios (ORs) and 95 % confidence intervals (CIs) were calculated using logistic regression. Restricted cubic spline regression was applied to evaluate the shape of the relationship between BAZ and primary dental caries.

**Results:**

The prevalence of primary dental caries was 30.7 % in total sample. Regarding dmft values, the mean ± standard deviation (SD) in the combined sample were 1.03 ± 2.05 in boys and 0.93 ± 1.92 in girls. Both indices decreased by age. Compared with normal BMI group, children in overweight and obesity groups have 27 % (OR = 0.73, 95 % CI: 0.66-0.81, *P* < 0.0001) and 34 % (OR = 0.66, 95 % CI: 0.59-0.74, *P* < 0.0001) lower odds for the presence of primary dental caries after adjustment for age and gender, respectively. Although in general, increased BAZ was associated with decreased risk of dental caries, full-range BAZ was associated with dental caries in an A-shaped manner with a zenith at around -1.4.

**Conclusion:**

Higher BMI was associated with lower odds of caries; overweight and obese children were more likely to be primary dental caries free among 7-9 years in Guangzhou, China.

**Electronic supplementary material:**

The online version of this article (doi:10.1186/s12889-016-3295-3) contains supplementary material, which is available to authorized users.

## Background

Dental caries remains the most commonly encountered chronic disease of childhood, even though it is largely preventable [1]. According to the report of World Health Organization (WHO), a total of 60-90 % school children worldwide have experienced caries, with the disease being most prevalent among Asians and Latin Americans [2]. In China, the Third National Oral Health Epidemiologic Survey in 2005 reported that in 5 years old school children, the prevalence of primary dental caries was 66 % and 3.5 teeth per person were either decayed, missing or filled among that age group [3]. Poor oral health is detrimental to children since it affects their nutrition, growth and development. Thus, the need for risk factor identification and immediate preventative action for dental caries in children is warranted.

Oral health is strongly influenced by the daily intake of food such as energy-dense foods and drinks, which also play a vital role in the development of obesity. The prevalence of childhood obesity has also increased dramatically in many countries around the world, with China being no exception. In China, children and adolescents were diagnosed as overweight/obesity increased from 1.8 % and 0.4 % respectively in 1981–1985 to 13.1 % and 7.5 % respectively in 2006–2010 [4]. Although an increased caries experience is biologically plausible in obese/overweight children, previous individual studies exerted to determine the associations between body mass index (BMI) and dental caries have reached inconsistent findings. Some studies showed that dental caries was related with high BMI [5–11], while others demonstrated an inverse association [12–20] or no relationship [21–26]. A recent review has reported a weak to moderate correlation (*r* = 0.40) between increasing obesity prevalence and increasing caries prevalence [27], but in another systematic review of longitudinal studies, agreement has not been reached because of the varied associations shown in the included studies [28]. Nevertheless, most previous studies were conducted in Western countries and Indian, and had limited sample size (less than 1,000) or focused on certain age group. Scarce evidence has been produced to link dental caries and BMI in Chinese children in a relatively wide age range, who are thought to exhibit entirely different dietary habits and lifestyles. Therefore, more researches are needed to address these important public health problems to facilitate prevention and health promotion.

In this study, we aimed to investigate the oral health status and its association with BMI in Guangzhou elementary school children aged 7-9 years old with a large sample size.

## Methods

### Subjects enrollment

The data were derived from the surveys on students' constitution and health carried out by the government in 2011, in Guangzhou, Guangdong Province, China. Through a multistage cluster sampling, we first randomly selected 65 schools from the urban area, and in a second stage, students aged 7–9 years from those schools were invited to participate in this survey. Among 36,555 students, 33959 (92.9 %) pupils were recruited and written informed consent was obtained from the parents of all children who participated in the survey. Approval for the study was obtained from the Ethics Committee of the School of Public Health, Sun Yat-Sen University (NO.201414). All subjects suffering from obvious diseases (*n* = 335, like hepatitis, tuberculosis, etc.) or physical/mental deformities (*n* = 146, e.g., physical disability or malformation, eating disorder) and missing anthropometric data (*n* = 917, absence from examination) were excluded. The parents of the excluded children also provided written informed consent. Finally, 32,461(88.8 %, 14,778 girls and 17,683 boys)students were included in this cross-sectional study.

### Anthropometric measurement

Weight and height were measured with participants dressed in light clothing and barefoot. Body weight was measured to the nearest 0.5 kg with a digital scale. The height was measured to the nearest 0.5 cm with a portable measuring unit. Each parameter was measured twice and a third measurement was carried out if the difference was ≥ 2 cm. BMI was calculated as weight (in kilograms)/height (in meters) squared. The subjects were categorized as underweight, normal weight, overweight, and obesity according to the sex- and age-stratified cutoff values of BMI recommended by Group of China Obesity Task Force in 2003 and the reference norms established from the 2010 national physical fitness and health surveillance data [29]. Underweight is defined as under the 5th percentile curve, normal weight as between 5th and 85th percentile, overweight as higher than 85th and lower than the 95th percentile and obesity as higher or equal to the 95th percentile. We also applied the criteria recommended by WHO [30], Centers for Disease Control and Prevention (CDC) [31] and World Obesity/Policy & Prevention (formerly IOTF) [32] to categorize the children to the aforementioned four BMI subgroups.

Previous studies have documented that partitioning the continuous BMI score into four groups might result in less information being carried by the data, a reduction or spurious increase in statistical power, with resultant Type I or Type II errors [23, 33]. Additionally, a recent systematic review showed a significant association between obesity and caries according to Z-score of BMI-for-age (BAZ), but the findings were non-significant when BMI was used [34]. Hence, we transformed BMI into BAZ using LMS method to further validate the relationship. The BAZ was calculated as: Z = ((X/M)^L-1)/L/S [35] and the L, M, S parameters for the calculation were provided by the 2007 WHO Growth Reference [30].

### Dental caries assessment

Full mouth dental caries detection was performed by eight trained doctors, of whom two were senior dentists and the others were physicians, with reference to the WHO criteria [36]. It took two to three minutes for each child. The kappa agreement value for inter-examiners was 0.80. Caries was recorded as present when a lesion in pit or fissure, or on a smooth tooth surface, has a detectably softened floor, undermined enamel, or softened wall. If restoration materials were present, the teeth were also included in this category. The caries index was measured as the number of decayed (d), missed (m) and filled (f) teeth (t), the mean dmft index for primary dentition.

### Statistical analysis

Data input was performed using Epidata 3.1 software. Variables were expressed as mean ± standard deviation (SD) or percentage. Chi square test was applied to compare the prevalence of dental caries across different groups stratified by age, gender or weight. For evaluation of the differences of dmft between two groups, Mann-Whitney U test was performed, and for three or more groups, Kruskal-Wallis test was used. Logistic regression models were performed to analyze the association between BMI and the risk for the prevalence of primary dental caries after adjustment for age in sex stratified analysis and for age and sex in the combined sample. Odds ratio (OR) and their 95 % confidence intervals (95 % CI) were calculated. We further fitted a logistic regression model with restricted cubic splines using BAZ as a continuous variable to explore potential nonlinear association [37]. The reference value was 0 and the five knots assigned were -2, -1, 0, 1, and 2. A spline function was assumed to be significant if the *P*-value for the model *χ*^*2*^ was ≤0.01. Tests for linearity used the Wald test. Statistical analysis was carried out using SPSS Statistics 19.0 Software (SPSS, Inc., Chicago, USA) and Stata Software (version 13.1; Stata Corp, College Station, TX). A *P* value < 0.05 was considered statistically significant.

## Results

A total of 32,461 pupils (17,683 boys and 14,778 girls) aged 7 to 9 years were enrolled in the present data. The gender and age-specific BMIs are presented in Table 1. BMI increased with age regardless of sex in general.

**Table 1:** The number of children and their BMI stratified by age and sex

Age(y)	BMI (Mean ± SD)					
	*N*	Boys	*N*	Girls	*N*	Overall
7	5892	15.7 ± 2.1	4825	14.9 ± 1.7	10717	15.4 ± 2.0
8	5942	16.3 ± 2.5	5060	15.4 ± 2.1	11002	15.9 ± 2.4
9	5849	16.9 ± 2.9	4893	16.0 ± 2.4	10742	16.5 ± 2.7
Overall	17683	16.3 ± 2.6	14778	15.4 ± 2.1	32461	15.9 ± 2.4

BMI: body mass index; SD: standard deviation

Table 2 depicts the gender and age-stratified prevalence of primary dental caries and mean (SD) of dmft. In boys, the prevalence of primary dental caries and mean (SD) dmft value respectively decreased from 32.8 % and 1.21 (2.38) at 7 years of age to 29.3 % and 0.84 (1.69) at 9 years of age. The corresponding values in girls decreased from 31.4 % to 26.5 % and from 1.10 (2.18) to 0.71 (1.51), respectively. In the combined sample, the prevalence for primary dental caries was 30.7 %, which in boys was significantly higher than in girls (31.4 % vs. 29.7 %, *P* < 0.001). The mean dmft was also significantly higher in boys than in girls (1.03 ± 2.05 vs. girls 0.93 ± 1.92, *P* < 0.001).

**Table 2:** Prevalence of primary dental caries and mean dmft value by age and sex

Age(y)	Dental caries *N*(%)				dmft (mean ± SD)			
	Boys	Girls	*P* ^*a*^	Overall	Boys	Girls	*P* ^*b*^	Overall
7	1934 (32.8)	1514 (31.4)	0.112	3448(32.2)	1.21 ± 2.38	1.10 ± 2.18	0.0667	1.16 ± 2.29
8	1910(32.1)	1583(31.3)	0.334	3493(31.8)	1.05 ± 2.00	1.00 ± 1.97	0.219	1.03 ± 1.97
9	1715(29.3)	1296(26.5)	0.0011	3011(28.0)	0.84 ± 1.69	0.71 ± 1.51	<0.001	0.78 ± 1.61
Overall	5559(31.4)	5982(29.7)	<0.001	9952(30.7)	1.03 ± 2.05	0.93 ± 1.92	<0.001	0.99 ± 1.99
*P* ^*c*^	<0.0001	<0.0001	—	<0.0001	<0.0001	<0.0001	—	<0.0001

dmft : the number of decayed (d), missed (m), and filled (f) teeth(t) for primary teeth

SD: standard deviation

^a^analyzed by Chi-Square test

^b^analyzed by Mann-Whitney U test

^c^ *P* for dental caries was analyzed by Chi-Square test, and *P* for dmft was analyzed by Kruskal-Wallis Test

As listed in Table 3, based on the criteria in China, about 64.2 %, 15.4 %, 11.7 %, 8.8 % boys were categorized as normal weight, underweight, overweight and obesity, respectively. The corresponding estimations in girls were 71.1 %, 17.4 %, 7.6 %, 3.9 %, respectively. In boys and girls as well as the total sample, the number of primary dental caries and the dmft decreased with increasing BMI. The percentages of children classified to the four BMI subgroups according to the criteria of WHO, CDC or IOTF are showed in Additional file 1: Table S1, S2 and S3.

**Table 3:** Prevalence of primary dental caries and mean dmft values in each BMI subgroup based on the criteria in China

BMI Groups	*N* (%)	Primary dental caries *N*(%)	dmft (Mean ± SD)
Boys			
Normal weight	11354(64.2)	3693(32.5)	1.08 ± 2.09
Underweight	2717(15.4)	928(34.2)	1.20 ± 2.27
Overweight	2064(11.7)	560(27.1)	0.81 ± 1.73
Obesity	1548(8.8)	378(24.4)	0.68 ± 1.52
*P* ^*a*^	—	<0.0001	<0.0001
Girls			
Normal weight	10514(71.1)	3204(30.5)	0.96 ± 1.93
Underweight	2565(17.4)	782(30.5)	0.99 ± 2.04
Overweight	1128(7.6)	272(24.1)	0.67 ± 1.57
Obesity	571(3.9)	135(23.6)	0.64 ± 1.47
*P* ^*a*^	—	<0.0001	<0.0001
Total			
Normal weight	21868(67.4)	6897(31.5)	1.02 ± 2.01
Underweight	5282(16.3)	1710(32.4)	1.10 ± 2.17
Overweight	3192(9.8)	832(26.1)	0.76 ± 1.67
Obesity	2119(6.5)	513(24.2)	0.67 ± 1.51
*P* ^*a*^	—	<0.0001	<0.0001

dmft : the number of decayed (d), missed (m) and filled (f) teeth(t) for primary dentition

BMI: body mass index; SD: standard deviation

^a^: *P* for dental caries was analyzed by Chi-Square test, and *P* for dmft was analyzed by Kruskal-Wallis Test

**Fig. 1:**
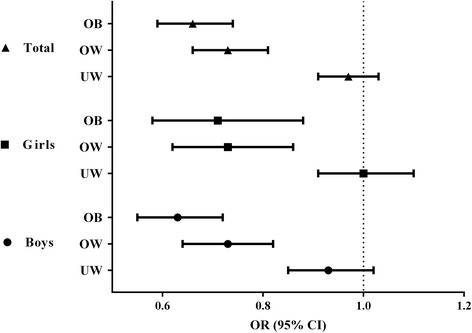
The odds ratio (95 % CI) for the prevalence of primary dental caries in obesity, overweight and underweight children using normal weight group as reference. Children were classified to the four BMI subgroups based on the criteria in China. OB: Obesity, OW: Overweight, UW: Underweight. In each gender, age was applied for adjustment; in the whole sample, age and sex were used as covariates

The association of the dental caries prevalence and BMI are demonstrated in Fig. 1. Using normal BMI groups as a reference, the odds for the prevalence of primary dental caries decreased by 27 % (OR = 0.73, 95 % CI: 0.66-0.81) and 34 % (OR = 0.66, 95 % CI: 0.59-0.74) in overweight and obese children after adjusting for age and sex, respectively. Similar associations were observed in both sexes when controlled by age. No statistically significant difference was identified in underweight groups. When we categorized the children to the four BMI subgroups based on the criteria recommended by WHO, CDC and IOTF, similar results were observed (see Additional file 1: Figure S1, S2 and S3).

**Fig. 2:**
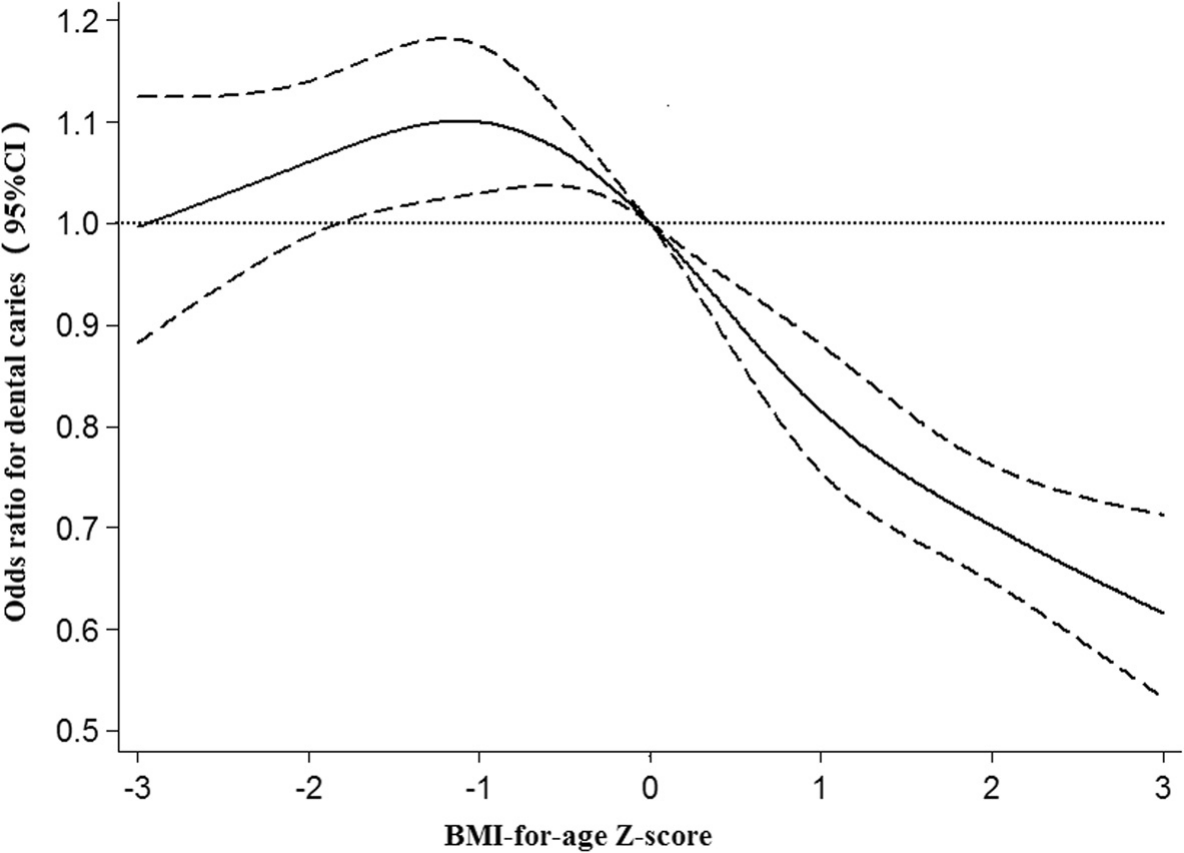
Restricted cubic spline regression for the association between BMI-for-age Z-score and the odds for the prevalence of primary dental caries. Solid line, odds ratio; dashed line, 95 % confidence interval. Knots were placed at Z-score = -2,-1, 0, 1, 2. The reference value was Z-score = 0. The model was adjusted age and sex

To illustrate the non-linear associations between BAZ and the odd of dental caries, we graphed the ORs and 95 % CI from models using restricted cubic splines for the total sample (Fig. 2). BAZ was significantly associated with dental caries in an A-shaped manner with a zenith at around BAZ = -1.4 and a long tail on the right (*P* < 0.0001). The *P-*value for the linearity was *P* < 0.0001. Children with BAZ > 1 were associated with reduced risk of dental caries, which remained significant after adjusting for sex and age.

## Discussion

Based on this large population-based cross-sectional study in Chinese children aged 7-9 years, we found the following main results: 1) the total prevalence of primary dental caries was 30.7 %; 2) the prevalence of primary dental caries decreased with age; 3) there was an inverse association between dental caries and BMI, in comparison with normal weight group, overweight and obese children were more likely to be primary dental caries free.

### The prevalence of dental caries

Bagramian et al. have documented that temporal trends showed a global decline in dental caries since the 1970s, yet recent data indicate an increase of the disease in both the primary and permanent dentition [38]. Based on the data from the United States National Health and Nutrition Examination Survey (NHANES), the prevalence of primary dental caries has remained unchanged (49.90 % in 1988–1994 vs. 51.17 % in 1999–2004, *P* > 0.05) among youths aged 6–11 years [39]. According to the report on The Physical Fitness and Health Research Of Chinese Students in 2005, the prevalence of primary dental caries and mean (SD) of dmft were 60.9 % and 3.56 (4.65) in children aged 5 years in urban areas in Guangdong, China, respectively. Both indices declined compared with the report in 1995 (76.55 % and 4.48, respectively) [3]. Similar to the secular trend, a further reduction for caries prevalence was indicated in our study in comparison to the results in 2005, which presented a 0.5-fold reduction (30.7 % vs. 60.9 %). The prevalence of dental caries in our data was relatively low, and the following reasons might be involved: firstly, we only examined the children in urban areas in Guangzhou. Higher living standard and better educational attainment improved children’s access to dental care and the availability of fluoride-containing products; secondly, China's one child policy has turned many children into the “Little Emperor” of their families. The parents paid more attention to their child’s health status including oral health.

### The associations between dental caries and BMI

Caries is usually regarded as a consequence of frequent ingestion of fermentable sugars, which can also lead to obesity. As a result, the link between caries and obesity has long been suspected. However, research results into the obesity-caries relationship in children have been mixed and inconclusive. In the data of NHANES III, Kopycka-Kedzierawski et al. suggested that overweight may be associated with decreased rates of caries in children aged 2-18 years [16]. Likewise, in a sample of 1951 in Philippine children aged 11-13 years, Benzian et al. also reported that there was a significant inverse association between caries and BMI and particularly between odontogenic infections and below-normal BMI [14]. A cross-sectional study conducted by Alkarimi et al. in Saudi Arabian children age 6-8 years old suggested an inverse linear association between dental caries and anthropometric outcomes [20]. A recent Chinese study among 744 8-year-old children also reported a weak negative association between caries severity and weight status [40]. In accordance with those findings, we also found that caries-free children were more overweight and obesity. However, in the study performed by Willerhausen et al. the authors found a significant positive association between weight and primary caries after adjusting for age in 1290 pupils aged 6-11 years [8]. Similarly, Marshall et al. reported that the greatest proportion of dental caries was found in the group of obese subjects in a sample of 413 children [6]. Other studies with limited sample size (less than 1,000) have demonstrated no correlation between BMI categories and the prevalence of dental caries [21, 23–26]. The heterogeneities between studies might be attributed to: 1) disparity of sample size. Most previous study sample sizes were less than 1,000. Nevertheless, our findings agreed with the results from national surveys in US [16]; 2) non-uniformity of the criteria for BMI classification across studies. Some studies used the CDC centiles [6, 9, 13, 23, 26, 40], other studies employed the international age and gender appropriate data sets recommended by the IOTF [5, 10, 12] or WHO [14, 19, 20, 22]. And in the current paper, we applied the thresholds developed by Group of China Obesity Task Force [29] and also the above mentioned three criteria for evaluation, and we found that the results were similar; 3) different indices and definitions of caries. For example, some studies reported dmft, whereas others reported dmfs (decayed, missing, filled, surfaces); 4) variation in the confounders used for adjustment such as sex, race/ethnicity, socioeconomic and nutritional status. Underweight children are usually under-nutrition. Enamel hypoplasia, salivary glandular hypofunction and saliva compositional changes may be mechanisms through which malnutrition is associated with caries as reviewed by Psoter et al. [41]. And in turn, untreated caries might have caused severe pain and discomfort in children and, thus, reduced food intake. In addition, other symptoms induced by caries including infection, irritability, and disturbed sleeping habits can affect children's quality of life and thereby growth [42]. A longitudinal design will be helpful in understanding the course and consequences relationship.

### Strength and limitation

The strengths of this study include a relatively large sample size, wide age range, and inclusion of both sexes and the use of a standardized diagnostic interview administered by trained interviewers. Also, we used restricted cubic spline regression to evaluate the shape of the relationship between BAZ and primary dental caries. Some potential limitations also warrant discussion. First, no causal association can be made as with any cross-sectional study. Second, dental caries detection was carried out visually and no X-rays were taken, thus the prevalence of dental caries and dmft value might be underestimated. Thirdly, other factors including energy-dense foods and drinks, family/parental socioeconomic status, vitamin D have also been observed to exert significant influences on the presence of dental caries and obesity as well [20, 43]. However, we have not evaluated those confounders in our data. Therefore, residual confounding could bias the observed associations. Finally, since the sample referred in urban Guangzhou citizens, more work is needed to evaluate whether these results extrapolate to rural samples and other racial or ethnic groups.

## Conclusion

In conclusion, our results suggested that children at risk of being overweight and obesity generally had lower caries experience than their normal weight peers in urban Guangzhou, China. Further longitudinal studies are required to confirm the results of the present study, and to determine the mechanisms of the association between BMI and dental caries.

## Abbreviations

BMI, Body Mass Index; WHO, World Health Organization; DMFT, Decayed Missing or Filled Teeth; BAZ, BMI-for-Age Z-score; OR, Odds Ratios; CI, Confidence Interval; SD, Standard Deviation; CDC, Center for Disease Control and Prevention; IOTF, World Obesity/Policy & Prevention; NHANES, National Health and Nutrition Examination Survey

## Additional file

Additional file 1:
**Table S1**. Prevalence of primary dental caries and mean dmft values in each BMI subgroup based on the criteria of WHO. **Table S2**. Prevalence of primary dental caries and mean dmft values in each BMI subgroup based on the criteria of IOTF. **Table S3**. Prevalence of primary dental caries and mean dmft values in each BMI subgroup based on the criteria of CDC. **Figure S1**. The odd ratios (95 % CI) for the prevalence of primary dental caries in obesity, overweight and underweight children using normal weight group as reference. Children were classified to the four BMI subgroups based on the criteria of WHO. **Figure S2**. The odd ratios (95 % CI) for the prevalence of primary dental caries in obesity, overweight and underweight children using normal weight group as reference. Children were classified to the four BMI subgroups based on the criteria of IOTF. **Figure S3**. The odd ratios (95 % CI) for the prevalence of primary dental caries in obesity, overweight and underweight children using normal weight group as reference. Children were classified to the four BMI subgroups based on the criteria of CDC. (DOCX 141 kb)
