# Supplementary material for: Dental caries is negatively correlated with body mass index among 7-9 years old children in Guangzhou, China
Source: BMC Public Health. 2016 Jul 26;16:638. doi: 10.1186/s12889-016-3295-3 (PMC4960882; doi:10.1186/s12889-016-3295-3)
Supplement: Additional file 1: — Table S1. Prevalence of primary dental caries and mean dmft values in each BMI subgroup based on the criteria of WHO. Table S2. Prevalence of primary dental caries and mean dmft values in each BMI subgroup based on the criteria of IOTF. Table S3. Prevalence of primary dental caries and mean dmft values in each BMI subgroup based on the criteria of CDC. Figure S1. The odd ratios (95 % CI) for the prevalence of primary dental caries in obesity, overweight and underweight children using normal weight group as reference. Children were classified to the four BMI subgroups based on the criteria of WHO. Figure S2. The odd ratios (95 % CI) for the prevalence of primary dental caries in obesity, overweight and underweight children using normal weight group as reference. Children were classified to the four BMI subgroups based on the criteria of IOTF. Figure S3. The odd ratios (95 % CI) for the prevalence of primary dental caries in obesity, overweight and underweight children using normal weight group as reference. Children were classified to the four BMI subgroups based on the criteria of CDC. (DOCX 141 kb) [file 12889_2016_3295_MOESM1_ESM.docx]

Table S1 Prevalence of primary dental caries and mean dmft values in each BMI subgroup based on the criteria of WHO.

| BMI Groups | N (%) | Primary dental caries N(%) | dmft  (Mean±SD) | |
| --- | --- | --- | --- | --- |
| Boys |  |  |  |  |
| Normal weight | 11501(65.0) | 3797(33.0) | 1.11±2.12 |  |
| Underweight | 1923(10.9) | 662 (34.4) | 1.20±2.28 |  |
| Overweight | 2485(14.1) | 682(27.4) | 0.85±1.78 |  |
| Obesity | 1774(10.0) | 442(24.9) | 0.69±1.55 |  |
| *P^a^* | — | <0.0001 | <0.0001 |  |
| Girls |  |  |  |  |
| Normal weight | 11024(74.6) | 3344(30.3) | 0.96±1.94 |  |
| Underweight | 2080(14.1) | 640(30.8) | 1.00±2.05 |  |
| Overweight | 1042(7.1) | 265(25.4) | 0.71±1.59 |  |
| Obesity | 632(4.3) | 135(22.8) | 0.59±1.39 |  |
| *P^a^* | — | <0.0001 | <0.0001 |  |
| Total |  |  |  |  |
| Normal weight | 22525(69.4) | 7141(31.7) | 1.04±2.04 |  |
| Underweight | 4003(12.3) | 1302(32.5) | 1.10±2.16 |  |
| Overweight | 3527(10.9) | 947(26.9) | 0.80±1.72 |  |
| Obesity | 2406(7.4) | 577(24.0) | 0.67±1.52 |  |
| *P^a^* | — | <0.0001 | <0.0001 |  |

dmft : the number of decayed (d), missed (m) and filled (f) teeth(t) for primary dentition

WHO: Word Health Organization, BMI: body mass index, SD: standard deviation.

^a^: *P* for dental caries was analyzed by Chi-Square test, and *P* for dmft was analyzed by Kruskal-Wallis Test.

Table S2 Prevalence of primary dental caries and mean dmft values in each BMI subgroup based on the criteria of IOTF.

| BMI Groups | N (%) | Primary dental caries N(%) | dmft  (Mean±SD) | |
| --- | --- | --- | --- | --- |
| Boys |  |  |  |  |
| Normal weight | 9433(53.3) | 3139(33.3) | 1.12±2.13 |  |
| Underweight | 3547(20.1) | 1205(34.0) | 1.17±2.22 |  |
| Overweight | 3020(17.1) | 813(26.9) | 0.81±1.75 |  |
| Obesity | 1683(9.5) | 402(23.9) | 0.66±1.51 |  |
| *P^a^* | — | <0.0001 | <0.0001 |  |
| Girls |  |  |  |  |
| Normal weight | 7832(53.1) | 2403(30.7) | 0.98±1.95 |  |
| Underweight | 4391(29.7) | 1359(30.9) | 1.00±2.00 |  |
| Overweight | 1987(13.4) | 506(25.5) | 0.73±1.64 |  |
| Obesity | 568(3.8) | 125(22.0) | 0.56±1.34 |  |
| *P^a^* | — | <0.0001 | <0.0001 |  |
| Total |  |  |  |  |
| Normal weight | 17265(53.2) | 5542(32.1) | 1.05±2.05 |  |
| Underweight | 7938(24.5) | 2564(32.3) | 1.08±2.11 |  |
| Overweight | 5007(15.4) | 1319(26.3) | 0.78±1.71 |  |
| Obesity | 2251(6.9) | 527(23.4) | 0.63±1.47 |  |
| *P^a^* | — | <0.0001 | <0.0001 |  |

dmft : the number of decayed (d), missed (m) and filled (f) teeth(t) for primary dentition

IOTF : World Obesity/Policy & Prevention (formerly IOTF), BMI: body mass index, SD: standard deviation.

^a^: *P* for dental caries was analyzed by Chi-Square test, and *P* for dmft was analyzed by Kruskal-Wallis Test.

Table S3 Prevalence of primary dental caries and mean dmft values in each BMI subgroup based on the criteria of CDC.

| BMI Groups | N (%) | Primary dental caries N(%) | dmft  (Mean±SD) | |
| --- | --- | --- | --- | --- |
| Boys |  |  |  |  |
| Normal weight | 11960(67.6) | 3693(32.5) | 1.08±2.10 |  |
| Underweight | 2342(13.2) | 928(34.2) | 1.21±2.27 |  |
| Overweight | 1999(11.3) | 560(27.1) | 0.80±1.71 |  |
| Obesity | 1382(7.8) | 378(24.4) | 0.66±1.48 |  |
| *P^a^* | — | <0.0001 | <0.0001 |  |
| Girls |  |  |  |  |
| Normal weight | 10818(73.2) | 3282(30.3) | 0.96±1.92 |  |
| Underweight | 2746(18.6) | 833(30.3) | 0.98±2.02 |  |
| Overweight | 1018(6.9) | 228(22.4) | 0.63±1.49 |  |
| Obesity | 196(1.3) | 50(25.5) | 0.67±1.52 |  |
| *P^a^* | — | <0.0001 | <0.0001 |  |
| Total |  |  |  |  |
| Normal weight | 22778(70.2) | 7159(31.4) | 1.02±2.02 |  |
| Underweight | 5088(15.7) | 1643(32.3) | 1.09±2.14 |  |
| Overweight | 3017(9.3) | 761(25.2) | 0.74±1.64 |  |
| Obesity | 1578(4.9) | 389(24.7) | 0.66±1.48 |  |
| *P^a^* | — | <0.0001 | <0.0001 |  |

dmft : the number of decayed (d), missed (m) and filled (f) teeth(t) for primary dentition

CDC: Centers for Disease Control and Prevention, BMI: body mass index; SD: standard deviation.

^a^: *P* for dental caries was analyzed by Chi-Square test, and *P* for dmft was analyzed by Kruskal-Wallis Test.

Figure S1: The odd ratios (95%CI) for the prevalence of primary dental caries in obesity, overweight and underweight children using normal weight group as reference. Children were classified to the four BMI subgroups based on the criteria of WHO.

OB: Obesity, OW: Overweight, UW: Underweight, WHO: Word Health Organization.

In each gender, age was applied for adjustment; in the whole sample, age and sex were used as covariates.

Figure S2: The odd ratios (95%CI) for the prevalence of primary dental caries in obesity, overweight and underweight children using normal weight group as reference Children were classified to the four BMI subgroups based on the criteria of IOTF.

OB: Obesity, OW: Overweight, UW: Underweight, IOTF: World Obesity/Policy & Prevention (formerly IOTF).

In each gender, age was applied for adjustment; in the whole sample, age and sex were used as covariates.

Figure S3: The odd ratios (95%CI) for the prevalence of primary dental caries in obesity, overweight and underweight children using normal weight group as reference Children were classified to the four BMI subgroups based on the criteria of CDC.

OB: Obesity, OW: Overweight, UW: Underweight, CDC: Centers for Disease Control and Prevention.

In each gender, age was applied for adjustment; in the whole sample, age and sex were used as covariates.
